# Nitrogen dynamics in managed boreal forests: Recent advances and future research directions

**DOI:** 10.1007/s13280-015-0755-4

**Published:** 2016-01-07

**Authors:** Ryan A. Sponseller, Michael J. Gundale, Martyn Futter, Eva Ring, Annika Nordin, Torgny Näsholm, Hjalmar Laudon

**Affiliations:** Department of Ecology and Environmental Science, Umeå University, 901 87 Umeå, Sweden; Department of Forest Ecology and Mangement, SLU, Skogsmarksgränd, 901 83 Umeå, Sweden; Department of Aquatic Sciences and Assessment, Swedish University of Agricultural Sciences, 750 07 Uppsala, Sweden; Skogforsk, Uppsala Science Park, 751 83 Uppsala, Sweden; Department of Forest Genetics and Plant Physiology, Swedish University of Agricultural Sciences, 901 83 Umeå, Sweden

**Keywords:** Boreal forests, Clear cutting, Forest management, Nitrogen fixation, Plant–soil interactions, Watersheds

## Abstract

Nitrogen (N) availability plays multiple roles in the boreal landscape, as a limiting nutrient to forest growth, determinant of terrestrial biodiversity, and agent of eutrophication in aquatic ecosystems. We review existing research on forest N dynamics in northern landscapes and address the effects of management and environmental change on internal cycling and export. Current research foci include resolving the nutritional importance of different N forms to trees and establishing how tree–mycorrhizal relationships influence N limitation. In addition, understanding how forest responses to external N inputs are mediated by above- and belowground ecosystem compartments remains an important challenge. Finally, forestry generates a mosaic of successional patches in managed forest landscapes, with differing levels of N input, biological demand, and hydrological loss. The balance among these processes influences the temporal patterns of stream water chemistry and the long-term viability of forest growth. Ultimately, managing forests to keep pace with increasing demands for biomass production, while minimizing environmental degradation, will require multi-scale and interdisciplinary perspectives on landscape N dynamics.

## Introduction

Humans have dramatically altered the global nitrogen (N) cycle, with widespread and often divergent effects on ecological systems and environmental quality (Galloway et al. 2008). The accelerated production and use of N-based fertilizers over the last century has increased the productivity of terrestrial ecosystems, with positive effects on agricultural and forestry yields, human nutrition, health, and the economy at global scales (e.g., Townsend et al. 2003). At the same time, excess fertilizer use together with greater N emissions and loading contribute to a litany of well-documented and undesirable environmental changes, including loss of biodiversity (Bobbink et al. 2010), eutrophication of aquatic ecosystems (Conley et al. 2009), soil and surface water acidification (Moldan and Wright 2011), and reductions in air quality (Wolfe and Patz 2002). Thus, the critical challenge for both policy makers and land managers is to ensure sufficient plant available N in places where it can result in desired outcomes (e.g., increased yield), while at the same time prevent the accumulation and export of excess N to recipient systems where enrichment contributes to environmental degradation. Striking this balance requires a perspective on the N cycle that emphasizes how changing environmental conditions and land management activities impact terrestrial ecosystem processes and the exchanges with aquatic and atmospheric pools at multiple spatial and temporal scales. In this review paper, we explore these issues and challenges as they relate to N dynamics in managed boreal forest landscapes of Sweden.

Nearly 60 % of Sweden is covered by lowland boreal forests, with more than 95 % of this land committed to public or private forestry. The forest products industry in Sweden currently accounts for ~2 % of the annual gross domestic product (Swedish Forest Agency 2014), and goals to increase forestry yields have motivated changes in management that include expanding the area of managed lands, the use of different or genetically improved tree species, and/or more intense silvicultural and harvesting practices (Helmisaari et al. 2014). Concurrent to these changes, the potential threat of atmospheric N deposition to forest health (Binkley and Högberg 1997) and the broader environmental consequences of specific forestry practices have drawn concern from the public. In particular, the disturbance caused by clear cutting and the use of forest fertilization as a management tool can result in losses of terrestrial biodiversity (Nordin et al. 2005) and may increase the risk of N leaching to aquatic ecosystems, contributing to the eutrophication of the Baltic Sea (Conley et al. 2009). The need to better understand the economic and environmental tradeoffs associated with forest management has led to extensive basic and applied research to determine (1) how Sweden’s forests respond to increased N loading (e.g., Binkley and Högberg 1997; Gundale et al. 2014), (2) to what degree commonly used silvicultural practices (e.g., clear cutting) are actually related to the broader problem of eutrophication (e.g., Futter et al. 2010), and (3) how forestry-related activities may be carried out to promote tree growth, while minimizing unwanted effects of N enrichment (Ring et al. 2013).

In this review, we address key issues related to N dynamics of managed boreal forest landscapes, drawing largely from recent research conducted in the low atmospheric deposition region of northern Sweden and Finland. The primary goal is to integrate new, basic understanding of forest nutrient cycling with applied forestry research and watershed monitoring to discuss how managed forests respond to changing N inputs and in turn influence the storage and losses of N at multiple time scales. To facilitate this goal, we (1) highlight emerging themes related to plant nutrition and above–belowground interactions; (2) summarize our current understanding of ecosystem-level responses to changes in N loading; (3) explore the short- and long-term effects of forestry practices on the local and regional N balance; and (4) characterize the patterns, controls, and significance of hydrological losses of N. Finally, we identify key knowledge gaps in our understanding of N cycling in managed boreal forests and suggest avenues for future research.

## Refining the knowledge of N dynamics in Sweden’s forests

**Fig. 1:**
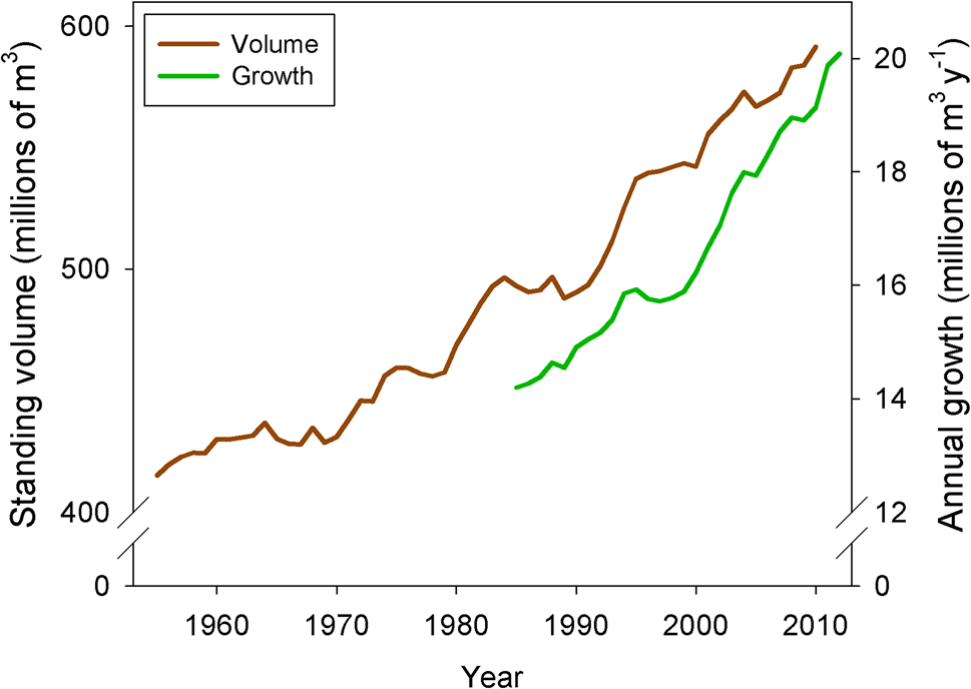
Total tree volume and annual growth increment for Västerbotten and Norrbotten counties in northern Sweden. Data are based on measurements from approximately 7000 national forest inventory (NFI) plots (Swedish NFI 1953–2012). Annual growth increment is based on a five-year moving average

**Fig. 2:**
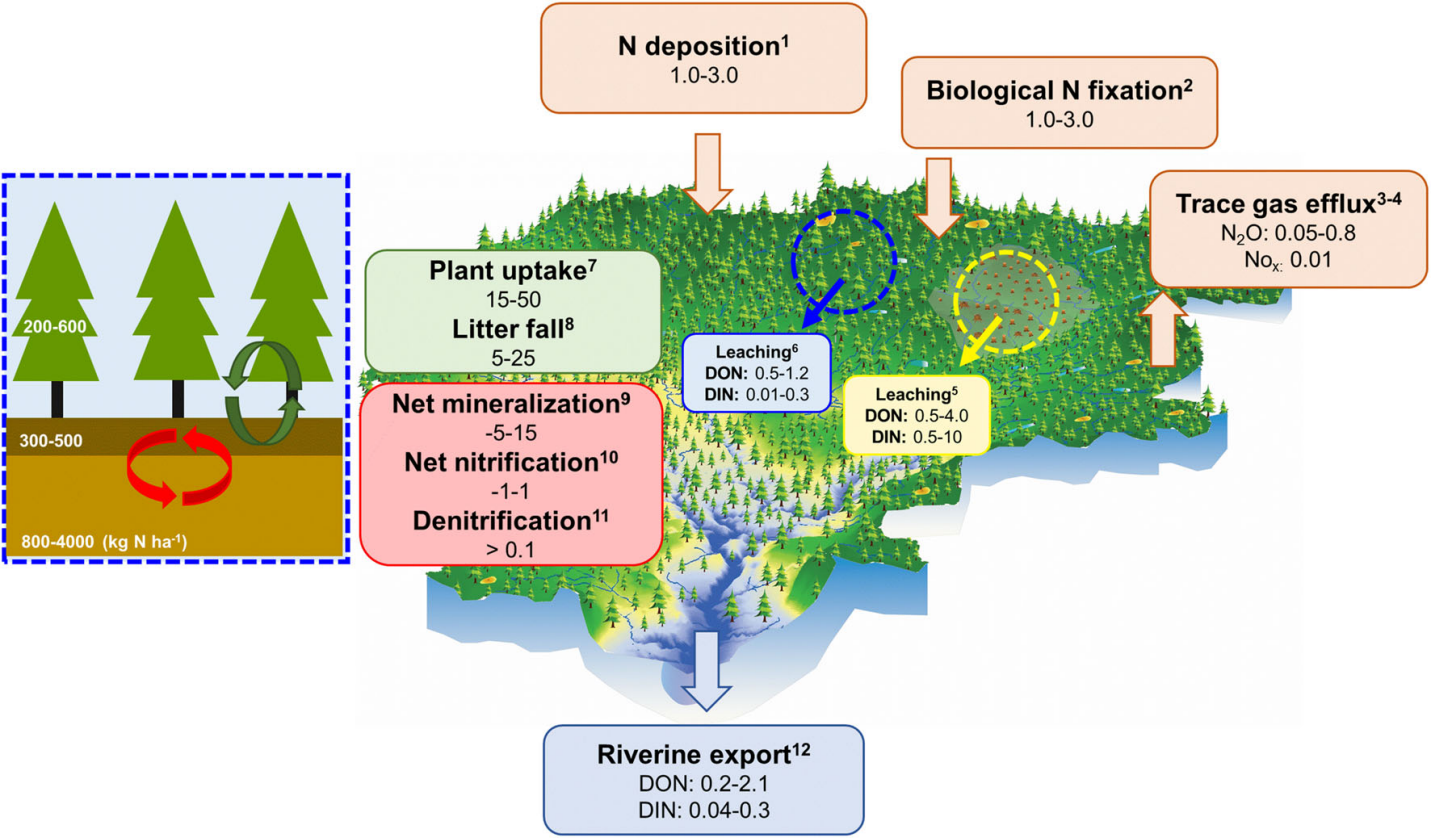
Summary of key pools and fluxes characteristic of a northern boreal watershed. Ranges for different N fluxes (kg ha^−1^ year^−1^) are based on literature values for northern Sweden and Finland wherever possible, and linked to the following references: *1* Gundale et al. 2011; *2* Lindo et al. 2013; *3* Kortelainen et al. 2006; *4* Korhonen et al. 2013; *5* Futter et al. 2010, Palviainen et al. 2014; *6* Mustajärvi et al. 2008; *7* Binkley and Högberg 1997; *8* Ukonmaanaho et al. 2008; *9* Gundale et al. 2011; *10* Olsson et al. 2012; *11* Nohrstedt et al. 1994; *12* Kortelainen et al. 1997, 2006. N_2_O fluxes (ref 3) are based on soils with C/N > 20. Blue and yellow circles represent rates of inorganic and organic N (DIN and DON) leaching from undisturbed and recently clear-cut forests, respectively. Rates of internal N cycling and export by forest stands can be highly variable in space and time, and the estimates for net nitrification and denitrification are particularly uncertain, but are very likely to be low in northern boreal forests. The forest inset provides ranges for N stocks in overstory, surface soils, and mineral soil compartments (see Egnell et al. 2015; Stuiver et al. 2015; Maaroufi et al. 2015)

Standing tree volume and growth in northern Sweden have increased by ~50 and ~40 % over the last century, respectively (Fig. 1; Swedish NFI, 1953–2012). Understanding how the N required to support this growth is maintained is a key question and challenge for managers. Inputs of N to boreal forest ecosystems occur mainly through biological N_2_ fixation and, during the last century, through deposition of atmospherically transported N of anthropogenic origin (Fig. 2). The latter exhibits a large variation across Sweden, ranging from 10–15 kg N ha^−1^ year^−1^ in the south to 1–3 kg N ha^−1^ year^−1^ in the north (Gundale et al. 2011). N_2_ fixation is more difficult to quantify and is chiefly governed by the activity of cyanobacteria living in association with feather mosses, which may contribute up to 3 kg N ha^−1^ year^−1^ in forests of northern Sweden (Lindo et al. 2013). In some cases, other plant species (e.g., *Alnus* spp. and *Lupinus* spp.) having symbiotic relationships with N_2_-fixing bacteria can represent important, additional sources of N to Swedish soils (Myrold and Huss-Danell 2003). However, given that the vast majority of Sweden’s boreal forests are dominated by Scots pine (*Pinus sylvestris*) and Norway spruce (*Picea abies*), the bulk of contemporary N_2_ fixation is associated with feather moss–cyanobacterial associations in understory communities (DeLuca et al. 2002). Regardless, given that growing boreal forest stands take up in the range of 15–50 kg N ha^−1^ year^−1^ (Binkley and Högberg 1997; Korhonen et al. 2013), the majority of N required to meet plant demand must be derived from litter and soil stocks accumulated over time.

Despite the fact that soils store between 1000 and 8000 kg N ha^−1^ in organic and mineral horizons (Egnell et al. 2015), plant growth in Swedish forests is with few exceptions N limited (Hyvönen et al. 2008). This limitation occurs because the bulk of soil N is bound in complex organic structures that are resistant to decay and too large for direct uptake by plants or microbes (Vitousek et al. 2002). Given this, the depolymerization of large organic N compounds into low molecular weight dissolved organic N (LDON; e.g., amino acids) through the action of extracellular enzymes is considered the key rate-limiting process in the forest N cycle (Schimel and Bennet 2004). Once produced, LDON may be taken up directly by plants (see below) or immobilized by bacteria and fungi, which can themselves be N limited in boreal soils (Näsholm et al. 2013).

When and where N demand is satisfied, soil microbes may metabolize (i.e., mineralize) LDON to meet energy demands, producing ammonium (NH_4_^+^). Net N mineralization rates tend to be comparatively low in boreal surface soil horizons (e.g., −5 to 15 kg N ha^−1^ year^−1^; Gundale et al. 2011; Olsson et al. 2012) where carbon (C) stores are large and N limitation strong, but may increase in mineral soils where greater microbial demand for C relative to N promotes this process (Wild et al. 2015). When produced, NH_4_^+^ may in turn be immobilized by plants and microbes, or further oxidized to nitrate (NO_3_^−^) via nitrification. Nitrification is significant to the ecosystem N balance because NO_3_^−^ is particularly mobile in soils and more readily subject to leaching losses. Moreover, the use of NO_3_^−^ as a terminal electron acceptor in denitrification can be an important source for N gas production and loss. However, like N mineralization, rates of net nitrification (and thus denitrification) are typically low in undisturbed, northern boreal forest soils (e.g., Högberg et al. 2006b; Olsson et al. 2012). Overall, the limited net production of inorganic N in these systems results in a fairly ‘closed’ N cycle at the scale of the forest stand, such that hydrological losses (Kortelainen et al. 1997) and trace gas fluxes (e.g., N_2_O; Klemedtsson et al. 2005) are typically small relative to inputs and internal cycling (Fig. 2).

Historically, the assumption has been that plant available N is represented primarily by the sum of inorganic N forms (NH_4_^+^ and NO_3_^−^); however, more recent studies in high latitude ecosystems show that plants can and do access a wide range of LDON compounds (Näsholm et al. 1998, 2009; Kielland et al. 2007). Despite this recognition, quantifying the relative importance of these sources is complicated by the very rapid turnover and inter-conversions of different N forms. Indeed, LDON compounds like amino acids and small peptides can exhibit turnover rates in soil of less than an hour (Jones and Kielland 2002). In addition, it is the diffusive flux of compounds, rather than total concentration, that is critical to plant uptake, and these rates are not captured by standard methods. Traditional soil sampling followed by extraction of sieved fractions with salt solutions runs the risk of *underestimating* the proportion of LDON relative to the total pool and *overestimating* the true availability of inorganic N (e.g., NH_4_^+^), which may be tightly bound in soils.

New techniques based on non-invasive, miniaturized diffusion probes (microdialysis) that capture the actively cycling N pool indicate that this underestimation of LDON can be severe in boreal soils (Inselsbacher and Näsholm 2012; Inselsbacher et al. 2014). Head-to-head comparison of these methods across a forest fertilization gradient showed that while NH_4_^+^ accounted for 80 % of the N pool following traditional extractions, microdialysis of the same soils revealed that amino acids were the dominant (80 %) fraction (Inselsbacher and Näsholm 2012). Moreover, the microdialysis approach showed large increases in LDON flux in response to external N loading that was not mirrored in the inorganic N pool. This result reinforces the importance of LDON as a plant source and suggests that the microdialysis approach effectively captures important variation in site fertility. Organic N sources have also been shown experimentally to stimulate growth of roots, fine roots, and mycorrhiza to a larger degree than inorganic N (Gruffman et al. 2012). Thus, the availability of these resources may have wide ranging effects on soil biota and processes and more research is needed to better understand both the production and fate of LDON in response to environmental change and various management activities.

The mechanisms maintaining N limitation in Swedish forests remain an active area of research. Competition between plants and soil microbes for available soil N is clearly one important mechanism influencing the structure of both plant and microbial communities. The microbial pool includes mycorrhizal fungi, living in a mutualistic relationship with plants, and other free-living microorganisms in direct competition with plants for resources. Studies have revealed a strong negative relationship between tree growth and abundance of ectomycorrhizal fungi (e.g., Högberg et al. 2003), which was traditionally interpreted to mean that extensively developed symbiosis with mycorrhizal fungi is vital for strongly N-limited trees. However, fungi are themselves N-rich organisms, and in N-poor soils their growth will inevitably immobilize a substantial fraction of the N they acquire. Thus, transfer of N from mycorrhizal fungi to their hosts will always be in competition with the use of that N for the growth of the fungi itself. In addition, under low internal N status, plants respond by increasing their allocation of C belowground. This stimulates the growth of mycorrhizal fungi, which in turn will lead to a further immobilization of N in soils. Through this feedback loop, mycorrhizal fungi could potentially worsen rather than alleviate tree N limitation in N-poor soils (Näsholm et al. 2013; Franklin et al. 2014), particularly during later successional stages (Clemmensen et al. 2015). Reevaluating long-standing views on the nature of plant–mycorrhizal interactions, including the significance of mycorrhizal fungi for soil N processing (Lindahl and Tunlid 2015) and the maintenance of N limitation, will aid in our understanding of how northern boreal forest growth will respond to both environmental change and forest management.

## Stand-scale responses to external N inputs

The N acquisition by plants and soil microbes connects the biogeochemical cycles of C and N, and this linkage has formed the basis for extensive research aimed at understanding how increases in atmospheric N deposition may influence forest C balances at northern latitudes. Results from some of this work have been controversial, suggesting that as much as 500 parts of C are sequestered per unit of N deposition (Holland et al. 1997; Magnani et al. 2007), which would account for a large portion of annual anthropogenic CO_2_ emissions (Schlesinger 2009). In contrast, several long-term N fertilization experiments in Sweden suggest a much lower quantity of C sequestered per unit of N added. For example, Högberg et al. (2006a) evaluated forest volume increase in response to 30 years of N application, and as recalculated by deVries et al. (2009) demonstrated that this increased volume equated to approximately 25 parts C per unit of N added. Hyvönen et al. (2008) evaluated the long-term (14–30 years) effects of high doses of N input (30–200 kg N ha^−1^ year^−1^) across 15 forest sites in northern Europe and also suggested that approximately 25 kg C kg^−1^ N were sequestered, with even lower rates (20 kg C kg^−1^ N) found in response to extremely high N loading (i.e., >150 kg  ha^−1^ year^−1^). Similarly, integrating results from Gundale et al. (2014) and Marroufi et al. (2015) indicate that mature Swedish spruce forests also accumulate a relatively low quantity of C (26 kg C kg^−1^ N) in response to chronic, low dose N additions that simulate the upper level N deposition rates in the boreal region.

There are multiple explanations for these more modest C assimilation rates estimated from long-term field experiments. Importantly, it is evident that trees do not serve as the dominant direct sink for external N inputs. This observation was made already in the 1980s using ^15^N-labeled tracers (Melin et al. 1983), and has been supported by numerous subsequent studies using similar isotopic techniques (Templer et al. 2012). More recently, Gundale et al. (2014) showed in a forest exposed to low levels of chronic N applications that mature spruce trees took up <10 % of external N inputs, with forest understory and in particular the soil humus layers serving as the largest N sink (~40 % of the N label explained by each of these pools).

In addition, these studies point to an important role of both the forest understory and soils in influencing how boreal forest ecosystems respond to N inputs. In particular, when external N inputs are relatively low (<3 kg N ha^−1^ year^−1^), feather mosses may serve as a strong N sink (Gundale et al. 2011, 2013). Because they take up nutrients directly into their leaves that have very slow litter decomposition rates (Lindo et al. 2013), feather mosses convert inputs of highly available inorganic N (e.g., atmospheric N deposition) into relatively stable insoluble soil organic N that is energetically costly for vascular plants to acquire (Lindo et al. 2013). At the same time, feather mosses are also the primary source for biological N_2_-fixation in boreal forests, which is performed by cyanobacteria that live between their leaves (Gundale et al. 2012; Lindo et al. 2013). Gundale et al. (2011, 2013) showed that N_2_-fixation by feather moss-cyanobacteria associations declines exponentially in response to N enrichment, with sharp (non-linear) reductions at low levels of input (from 0.5 to 1.0 kg N ha^−1^ year^−1^) that offset ~50 % of exogenous inorganic N inputs at deposition rates typical of the boreal region.

Finally, given the global significance of carbon stores in boreal soils, the impact of external N inputs on soil carbon storage and balance in northern forests has also received considerable attention (Janssens et al. 2010). For example, Maarouffi et al. (2015) showed that N additions caused soil C to accumulate at a rate of 10 kg C kg^−1^ of N added, which accounted for more than 1/3 of the total C sequestered in the forest. This result was consistent with a previous study by Högberg et al. (2006a) showing that chronic N fertilizer doses resulted in 11 kg C kg^−1^ N sequestered in soils (de Vries et al. 2006). One major point of emphasis has been that high levels of chronic N enrichment can have strong negative effects on soil microbial biomass and respiration rates, particularly for fungi (Janssens et al. 2010; Maaroufi et al. 2015). Such inhibition may enhance ecosystem C storage, and several explanations have been given for this response, including reduced belowground C allocation by plants to mycorrhizal fungi, direct inhibition of enzymes involved in lignin degradation, a decrease in soil pH, and a decrease in C availability to saprotrophs (Treseder 2008; Janssens et al. 2010). However, these observations are based largely on studies using experimental doses of N that greatly exceed even the highest rates of deposition observed in Sweden (e.g., >100 kg N ha^−1^ year^−1^). Studies that simulate more realistic deposition rates for this region (e.g., from ambient to 20 kg N ha^−1^ year^−1^), at least over short-time scales, show that increasing N availability within this lower range can actually enhance rates of soil respiration and C turnover (Hasselquist et al. 2012). These results reinforce the idea that soil microbes in these ecosystems can serve as important sinks that compete with trees for N (Näsholm et al. 2013). More generally, these findings highlight important non-linear relationships between external N inputs and ecosystem response in these managed forest ecosystems that deserve further study.

## Forest management and N dynamics of forest stands

There remains a disconnect between basic research focused on understanding N dynamics and balance in boreal forests and the applied knowledge needed to sustainably manage these ecosystems. In Sweden, even-aged forestry is the dominant silvicultural system culminating in the clear cutting of 80–130-year-old stands. Clear-cut areas typically range from 2 to 8 ha in size (Swedish Forest Agency 2014) and may comprise stem only harvesting (SOH) or whole-tree harvesting (WTH). For WTH, a large share of the tree tops and branches with needles are harvested in addition to stems, and therefore a significant portion of the N bound in plant tissues is removed from the site, rather than being recycled locally. Because residual detritus can act as a ‘slow release fertilizer,’ the removal of this material during WTH may lead to reduced rates of forest growth during the subsequent rotation period (Egnell and Ulvcrona 2015). One key question is whether the removal of plant-bound N at harvesting will be sustainable over multiple future rotation cycles. To address this question empirically, Merilä et al. (2014) calculated the balance of atmospheric N deposition and soil leaching losses for 15 Finnish forest stands to show that net N inputs over a typical rotation period are likely sufficient to replace N removed during SOH, but not WTH. Similar conclusions have been drawn for northern Sweden (Akselsson et al. 2007) and given trends of increasing tree volume and growth (Fig. 1), coupled with low and stable rates of N deposition (Lucas et al. 2013), the question remains as to whether or not exogenous replacement of N following harvest will persist into the future without greater application of fertilizer.

Given the significance of the N balance over the course of a rotation, it is important to understand and account for how forestry operations influence losses and inputs of N in managed stands. In Sweden, final felling is generally followed by mechanical site preparation, regeneration, pre-commercial and commercial thinnings, and in some cases N fertilization (discreet events adding about 150 kg N ha^−1^), all of which may alter the N balance at different time scales. Physical disturbance of soils, increases in pH, altered thermal regimes, and reduced plant demand at harvesting and site preparation all may create a short-term surplus in bioavailable N (e.g., Nohrstedt 2000) and elevated rates of N mineralization and nitrification (e.g., Smolander et al. 1998), but the magnitude and direction of these responses vary markedly among studies (Kreutzweiser et al. 2008). Such changes are often associated with increased NO_3_^−^ leaching from soils, which may be exacerbated by historical fertilization of the stand (Berdén et al. 1997, but see Ring et al. 2013). In northern boreal forests, soil NO_3_^−^ leaching losses in response to clear cutting can increase from very low levels (e.g., <0.1 kg N ha^−1^ year^−1^; Mustajärvi et al. 2008) to >10 kg N ha^−1^ year^−1^; however, elevated rates typically persist for only 3–10 years following this disturbance (e.g., Futter et al. 2010). Similar increases in dissolved organic nitrogen (DON) leaching losses may also be observed in response to clear cutting and site preparation (e.g., Smolander et al. 2001). In addition to these effects on N *losses* over the short-term, forestry operations may also affect the distribution of feather mosses and therefore N *inputs* via biological fixation over a more extended period of time (Stuiver et al. 2015). Given its clear significance, more research is needed to understand how forestry-induced soil disturbance, shifts in light, thermal, and moisture regimes govern rates of N_2_ fixation and its contribution to the overall N balance of managed boreal forest over the course of a rotation.

Soil disturbance and the residual organic matter left behind following different silvicultural measures are not always uniformly distributed within stands and this can generate important internal heterogeneity in N storage and turnover. For example, at the scale of a few meters, site preparation creates exposed, mixed, or inverted soils that generate patchiness in vegetation establishment and soil–water N concentrations, altering local source/sink N dynamics (Ring et al. 2013). Similarly, when using cut-to-length logging systems for SOH and WTH, logging residues are piled in ‘heaps’ that can affect leaching of NO_3_^−^ from the upper soil (Wall 2008). For example, higher NO_3_^−^ concentrations have been found in soil water collected from areas covered by logging residues than in uncovered soil (e.g., Staaf and Olsson 1994). The full effect of these residues on leaching remains unclear, as heaps simultaneously reduce water influx to the soil and affect vegetation establishment (Wall 2008; Hedwall et al. 2013). Further experimental studies are needed to understand the within-stand heterogeneity in N storage and loss that arises from different management operations as well as from decisions related to the handling of logging residues. Importantly, future work should include efforts to upscale these dynamics in space and time to better place responses to management within the context of the broader forest N balance and long-term site fertility.

## Watershed losses of N in boreal landscapes

The export of N in small streams reflects the residual N that is not taken up by plants, stored in soils, or lost via trace gas flux in terrestrial and riparian habitats. Streams thus integrate all of the terrestrial processes described in sections above, as these play out across the mosaic of stands, soil types, and topographic units connected via groundwater flow. Given strong connections to land, stream nutrient exports have long been used to interpret terrestrial biogeochemical cycles and monitor the effects of environmental change on surface water quality.

In northern boreal landscapes, N export in streams and rivers is generally in the range of 1–2 kg N ha^−2^ year^−1^ and the vast majority (~70–90 %) of this pool is comprised of DON (Kortelainen et al. 2006). Such a pattern is expected for systems draining strongly N-limited landscapes, as bulk DON is largely composed of compounds that are not readily bioavailable and thus not retained by plants and microbes on land (Hedin et al. 1995). Consistent with this idea, boreal forest watersheds tend to retain a very large fraction of the inorganic N they receive (upwards to 90–95 %; Kortelainen et al. 1997), even when exposed to moderately high levels of atmospheric deposition (e.g., up to 10 kg N ha^−1^ year^−1^; Dise and Wright 1995) or fertilization (Binkley and Högberg 1997). Inorganic N concentrations in streams of northern Sweden thus tend to be very low (e.g., <50 μg N l^−1^) unless the surrounding watershed is subject to agricultural and/or urban development (Sponseller et al. 2014), or recent forestry operations (e.g., final felling; Löfgren et al. 2009).

Final felling in boreal landscapes typically results in increased concentration and export of inorganic N in small streams (e.g., Löfgren et al. 2009), but the timing, magnitude, and duration of these responses vary across studies (Kreutzweiser et al. 2008). These losses reflect increases in soil N leaching described above, coupled with altered hydrological patterns following timber removal (e.g., elevated groundwater levels). Such changes can translate into stream concentrations far above pre-disturbance levels and export rates of inorganic N as high as 1–2 kg N ha^−1^ year^−1^ (Palviainen et al. 2014). The magnitude of these responses increases with the proportion of drainage area harvested, and studies in boreal landscapes suggest a 30% threshold in cover by clear-cut land is required to observe detectable changes in stream chemistry and export (Palviainen et al. 2014). Additional drainage features may also govern such responses: for example, intact riparian buffer zones can reduce the lateral transport of NO_3_^−^ to streams following harvests through plant uptake, immobilization by soil heterotrophs, or loss via denitrification (and N gas production). However, the efficiency of nutrient removal by buffer zones varies (Ranalli and Macalady 2010), and peat-rich riparian soils in boreal landscapes may serve as sources rather than sinks for inorganic N (Fölster 2000). The management of riparian zones to efficiently reduce inputs of terrestrially derived nutrients to small boreal streams requires further consideration and study. In particular, forestry operations occurring within near-stream ‘water recharge zones’ may have disproportionately strong effects on stream responses in these landscapes (Kuglerová et al. 2014).

While final felling can induce dramatic increases in inorganic N concentrations in small boreal streams, these effects appear to manifest over relatively small spatial and temporal scales. First, while streamwater responses to harvesting may persist for more than 10 years (Palviainen et al. 2014), the most dramatic changes in inorganic N concentrations are typically observed during the first 3–5 years (Jerabkova et al. 2011). After this phase of enrichment, there is typically an extended period during a rotation when inorganic N concentrations are low and can limit rates of biological processes in boreal streams (e.g., biofilm productivity; Burrows et al. 2015). In addition, streamwater responses to harvesting seem to be restricted to headwaters, such that elevated inorganic N concentrations are rarely observed in higher order streams in areas of active forestry (Schelker et al. 2015). Indeed, at broader spatial scales, leakage from forest harvesting likely accounts for only a trivial proportion (3%) of NO_3_^−^ loading to the Baltic Sea (Futter et al. 2010). However, clear cutting can also result in increased hydrologic losses of DON (Palviainen et al. 2014), which is unlikely to be rapidly processed (e.g., taken up or transformed biologically) in small streams. DON export following harvests in boreal landscapes probably deserves more attention and could potentially contribute to downstream eutrophication. Overall, empirical studies that address the potential for boreal streams and rivers to process and retain N are lacking and would strengthen our understanding of how terrestrial and coastal environments are connected.

**Fig. 3:**
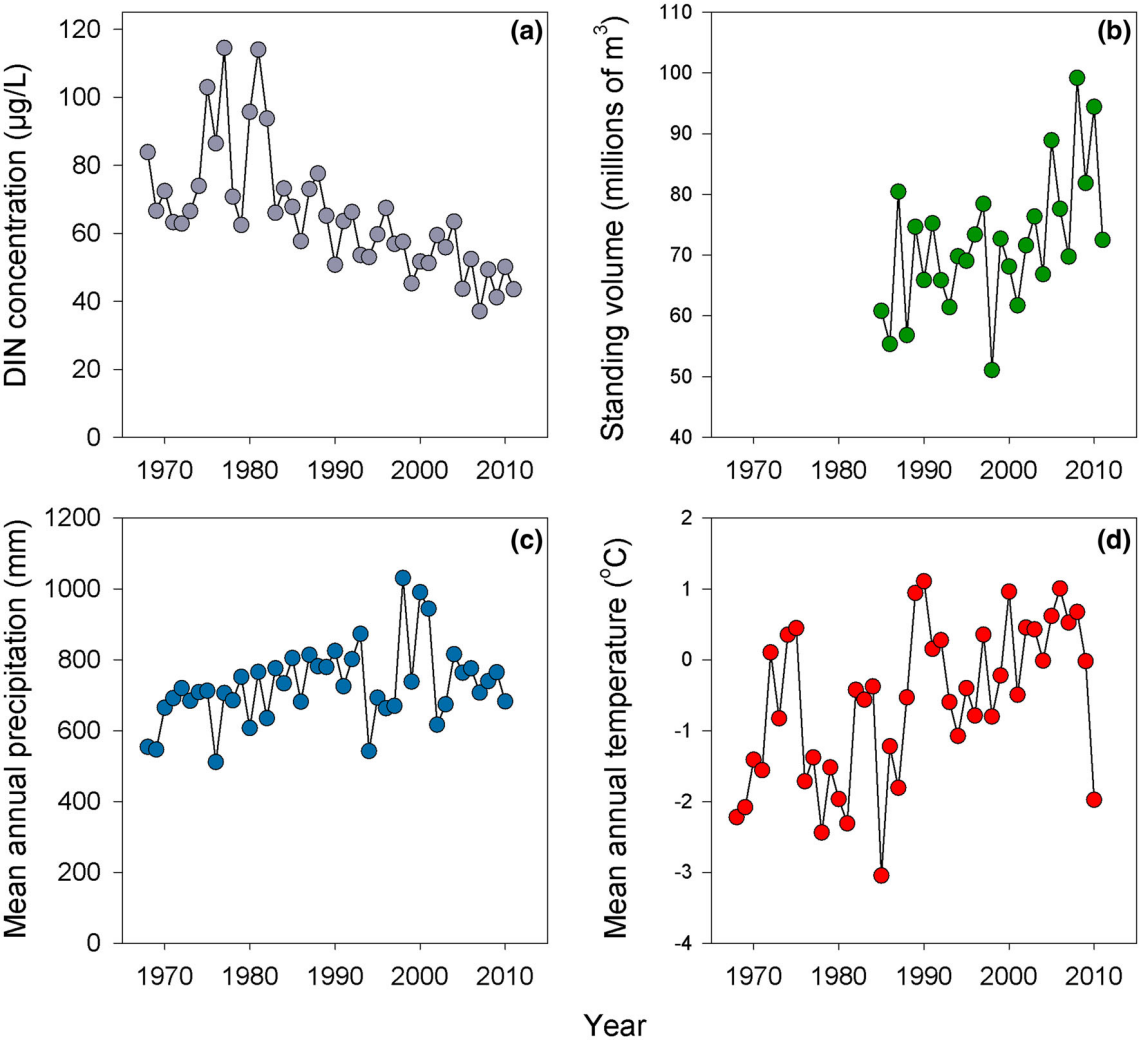
Long-term trends in **a** river inorganic nitrogen (DIN) concentration, **b** forest volume, **c** mean annual precipitation, and **d** mean annual temperature for the Vindeln River watershed of northern Sweden (10 000 km^2^). DIN represents the sum of nitrate and ammonium for samples collected monthly by the Swedish national monitoring program (Fölster et al. 2014). Forest volume was determined from national forest inventory (NFI) plots located within watershed boundaries (Lucas et al., 2016). Precipitation and temperature were estimated from the Hydrologiska Byråns Vattenbalansavdelning (PTHBV) database (from the Swedish Meteorological and Hydrological Institute, SMHI; Sponseller et al. 2014)

In contrast to these small scale responses to final felling, the long-term increases in forest biomass and growth across northern Sweden potentially have fundamentally different consequences for riverine export of DIN (Fig. 3). Recent assessment of long-term riverine nutrient records suggests declines in stream and river NO_3_^−^ concentrations across much of Sweden (Lucas et al. 2013). While these trends are most pronounced in southern Sweden, similar patterns were also observed for some northern systems. As an example, the average annual inorganic N concentration in the Vindeln River (a 10 000 km^2^ basin) has dropped by nearly 50 % since 1980 (Fig. 3a), with proportional declines in export (from ~0.2 to 0.1 kg N ha^−1^ year^−1^). These long-term declines observed in northern rivers are concurrent with increases in tree growth, standing volume, and accumulation of N in plant and soil biomass across the region (Fig. 3b; Lucas et al. 2016). Such patterns suggest a tightening of the terrestrial N cycle, and point to the potential for progressive N limitation (Luo et al. 2004) and increased terrestrial retention across a very broad spatial scale. The extent to which these long-term changes reflect advances in forest management and thus elevated tree growth, recovery from historical land use, increased protection of riparian buffer zones, or the effects of recent climate trends on terrestrial productivity (Fig. 3c, d) remains unknown and merits further study. Regardless, while reducing the delivery of nutrients to aquatic ecosystems is often a management goal, a continued reduction in the export of inorganic N may pose a challenge in northern Sweden, where the productivity of lakes (Bergström et al. 2008) and streams (Burrows et al. 2015) is already often N limited during the growing season.

## Future research directions

**Fig. 4:**
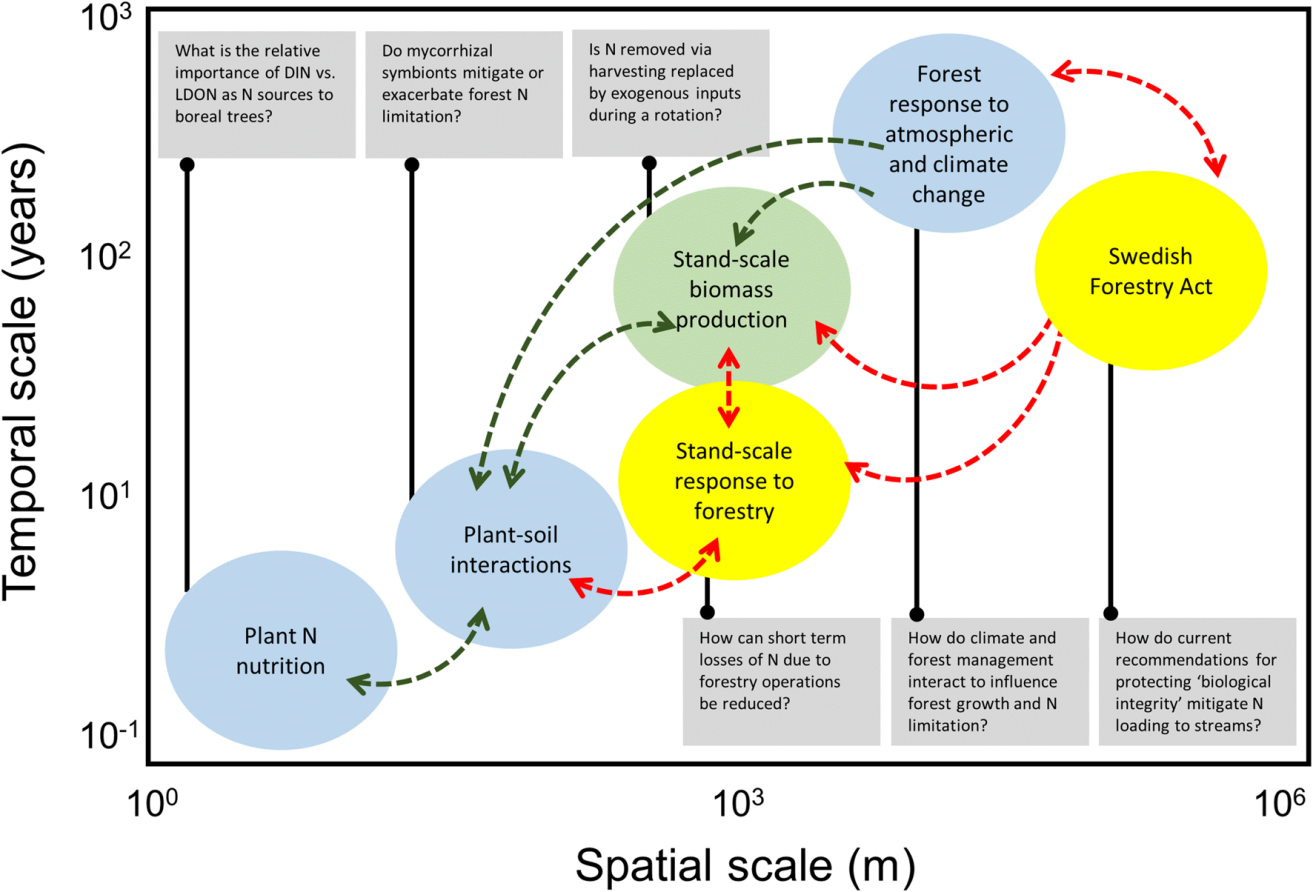
Stommel diagram summarizing key elements of N dynamics in boreal landscapes and their potential interrelationships across spatial and temporal scales. Stand-scale biomass production (*green*) represents the primary target of forest agencies and private landowners. This yield reflects ecological processes (*blue* with *green arrows*) related to tree N nutrition and a variety of plant–soil interactions that constrain N availability at small scales, as well as the constraints on growth imposed by broad-scale variation in climate and atmospheric N inputs. In addition, management-related decisions (*yellow* with *red arrows*) at the national scale drive local forestry operations that additionally influence ecosystem N dynamics, N losses to aquatic ecosystems, and tree growth. *Boxes* summarize key questions related to forest N cycling at each of these scales, as highlighted in the text

Current and historical forest management in northern Sweden has largely replaced other disturbance agents (e.g., fire) as the primary organizer of terrestrial ecosystem structure and function in the region (Gauthier et al. 2015). Forestry decisions and operations now govern the broad-scale distribution of stand developmental stages, which in turn dictate patterns of tree species composition, density, age-structure, and potential growth. This mosaic of forest stands provides the context within which soil N dynamics and above–belowground interactions operate at smaller scales. At the same time, it is the broader distribution of forest patches that responds to long-term variation in climate and atmospheric deposition to influence surface water quality and feedback onto regional- and global-scale biogeochemical cycles (Futter et al. 2016). Forest responses to climatic conditions in turn interact with management practices to determine the biomass yield at the end of a rotation. Thus, there are clear interconnections between basic ecosystem dynamics, applied forestry, and environmental quality that operate across a broad range of spatial and temporal scales in the Swedish landscape (Fig. 4). Despite these linkages, the research themes discussed above are embedded within different scientific traditions and have advanced independently, in some cases with little exchange of ideas. We argue that a greater focus on synthesizing existing basic and applied research on the response of forest ecosystems to land management and climate is needed to ensure sustainable forest management in the face of a changing climate and potential shifts in market demands for forest products.

Management aimed at promoting biomass production creates a form of ‘coerced resilience’ in the Swedish landscape, wherein inputs of resources and capital are required to maintain forest structures (e.g., in terms of stand density and age distributions) that are optimal for timber yield, but would otherwise not exist (Rist et al. 2014). Sustaining such systems requires a clear understanding of (1) the capacity for ‘natural supporting processes’ to maintain productivity at desired levels; (2) how this capacity may change in the long-term given continued or more intensive management; and (3) the extent to which effort to maintain production forests influences other ecosystems that serve as recipients for unwanted materials or waste. These requirements can only be achieved through communication and integration of ideas between scientists and managers working on related issues across levels of organization and ecosystem boundaries. Specifically, by promoting interaction among scientists engaged in plant biology, ecosystem ecology, soil science, hydrology, applied forestry, and environmental management, we see potential opportunities to achieve the following:

### Ensured long-term sustainability of forest growth

While evidence to date suggests that N removed during forest harvesting can ultimately be replaced via atmospheric deposition during a forest rotation (Merilä et al. 2014), it is important to determine whether this will continue to hold true in northern Sweden as forest growth increases, while N deposition remains low or declines, particularly if WTH methods are increasingly employed. As mentioned above, there is already some evidence that increased tree growth in northern Sweden could be linked with declines in hydrological exports of inorganic N (Lucas et al. 2016), suggesting greater N retention in terrestrial ecosystems and potentially a strengthening of forest N limitation. The possibility of increased N limitation as a result of forestry operations has clear implications for the sustainability of tree growth in the region. Exploring this problem requires continued experimental research on the controls over stand-scale N use and balance in response to different forestry operations, and over the course of forest regrowth. Such efforts should focus more on how above–below interactions influence plant nutrient acquisition over successional time and better incorporate potentially important biological vectors like N_2_-fixation and trace gas emissions. At the same time, a refined understanding of land–water interactions in boreal landscapes will allow us to better assess the degree to which riverine exports of nutrients capture meaningful trends in terrestrial ecosystem processes. Finally, while this review has focused exclusively on the significance of N, reports of declining tree mineral nutrition across Europe (Jonard et al. 2015) suggest that phosphorus or other elements may become limiting in the future.

### Increased forest growth with minimized N leaching

The potential for increased N limitation in northern forests could motivate greater use of fertilization as a management tool; however, such decisions and associated plans should be informed by the best possible science. For example, results from long-term enrichment experiments suggest that aboveground forest biomass is a comparatively weak sink for external inputs of inorganic N in the Swedish landscape (Gundale et al. 2013). A better understanding of what controls the ecosystem-scale efficiency of above- and belowground responses to N inputs has not only general implications for C–N interactions, but also for how to manage stands to attain desired yields, and for providing credible estimates of ‘critical loads’ for N (Nordin et al. 2005). Indeed, while forest fertilization does not always result in elevated inorganic N concentrations in outlet streams, we know that this can happen if fertilizer is applied over large areas, during periods of high discharge [e.g., >2000 % increase in stream NO_3_ was observed by Lundin and Nilsson (2014)]. Interdisciplinary efforts to maintain forest productivity (i.e., meet N demand), while reducing negative impacts on understory vegetation (e.g., loss of biodiversity), and minimizing leaching losses are clearly needed. Achieving this goal requires that we better understand the fate of externally supplied N to forests as well as how N demand (or potential efficacy of fertilizer application) varies with both climate and landscape context (see Laudon et al. 2016). Moreover, insights from research on the biology of plant nutrition (Näsholm et al. 2009) may lead to the development of management practices that stimulate more efficient N uptake by tree and/or of fertilizers and application techniques that more effectively deliver N to roots (Öhland and Näsholm 2002).

### Preparedness for global environmental change

Forest N dynamics interact closely with other biogeochemical cycles (e.g., carbon) and are further influenced by multiple climate changes, which may have particularly profound effects in the boreal region (Gauthier et al. 2015). Forest managers and policymakers will need to understand how landscape-scale N dynamics may respond to long-term environmental change and feedback to influence the future of forest growth. For example, while trends in increased standing biomass and productivity of northern Swedish forests (Fig. 1) are ostensibly linked to advances in silvicultural practices, they are also likely influenced by contemporaneous increases in atmospheric CO_2_, air temperature, and growing season length at northern latitudes (e.g., Lucas et al. 2016), all of which may serve to elevate terrestrial N demand (Luo et al. 2004). Such climate trends may also influence forest structure and nutrient dynamics through changes in patterns of vertebrate and invertebrate herbivory (Niemelä et al. 2001). Moreover, it is unknown how changes in tree growth and N demand will alter C allocation belowground to EMC fungi, which in turn may have feedback effects on nutrient availability and soil C dynamics. In addition to these ecosystem responses, expected shifts in soil freeze/thaw dynamics and hydrological patterns (Tetzlaff et al. 2013), together with potential modification of drainage systems (e.g., via ditch maintenance, stream crossing, and road construction), represent additional sources of uncertainty when predicting how climate trends may influence N cycling and runoff in the region. Predicting the consequences of climate change for forest N cycling—and assessing the long-term sustainability of forest growth and forest management—will require research on multiple fronts that asks how forest and hydrological responses to climate dynamics will alter plant N demand, soil N turnover, and lateral exports to downstream ecosystems.
